# Healthcare-based food assistance programmes in the United States: a scoping review and typology

**DOI:** 10.1017/jns.2023.111

**Published:** 2023-12-27

**Authors:** Rebecca K. Rudel, Elena Byhoff, Kiersten L. Strombotne, Mari-Lynn Drainoni, Jacey A. Greece

**Affiliations:** 1 Department of Community Health Sciences, Boston University School of Public Health, 801 Massachusetts Avenue, 4th Floor, Boston, Massachusetts 02118; 2 Department of Medicine, Section of Infectious Diseases, Boston University Chobanian & Avedisian School of Medicine/Boston Medical Center, 801 Massachusetts Avenue, 2nd Floor, Boston, Massachusetts 02118; 3 Department of Medicine, University of Massachusetts Chan Medical School, 55 Lake Avenue, North Worcester, Massachusetts 01655; 4 Department of Health, Law, Policy and Management, Boston University School of Public Health, 715 Albany Street, Boston Massachusetts 02118; 5 Evans Center for Implementation and Improvement Sciences, Department of Medicine, Boston University Chobanian & Avedisian School of Medicine, 801 Massachusetts Avenue, 2nd Floor, Boston, Massachusetts 02118

**Keywords:** Food assistance, Food insecurity, Healthcare-based intervention, Population health

## Abstract

This scoping review aimed to identify the breadth of healthcare-based food assistance programmes in the United States and organize them into a typology of programmes to provide implementation guidance to aspiring food assistance programmers in healthcare settings. We searched PubMed, Cochrane, and CINAHL databases for peer-reviewed articles published between 1 January 2010 and 31 December 2021, and mined reference lists. We used content analysis to extract programmatic details from each intervention and to qualitatively analyse intervention components to develop a typology for healthcare institutions in the United States. Eligible articles included descriptions of patient populations served and programmatic details. Articles were not required to include formal evaluations for inclusion in this scoping review. Our search resulted in 8706 abstracts, which yielded forty-three articles from thirty-five interventions. We identified three distinct programme types: direct food provision, referral, and voucher programmes. Programme type was influenced by programme goals, logistical considerations, such as staffing, food storage or refrigeration space, and existence of willing partner CBOs. Food provision programmes (*n* 13) were frequently permanent and leveraged partnerships with community-based organisations (CBOs) that provide food. Referral programmes (*n* 8) connected patients to CBOs for federal or local food assistance enrollment. Voucher programmes (*n* 14) prioritised provision of fruits and vegetables (*n* 10) and relied on a variety of clinic staff to refer patients to months-long programmes. Healthcare-based implementers can use this typology to design and maintain programmes that align with the needs of their sites and patient populations.

## Introduction

Food insecurity impacts approximately one in eight Americans.^(1)^ People who experience food insecurity lack reliable, consistent access to a sufficient quantity of food to live a healthful life.^(2)^ Households that experience low income are more likely to experience food insecurity and hunger than those with average incomes.^(3)^ While food insecurity is a social issue that can be addressed in multiple settings, evidence points to the connection between food insecurity and negative health outcomes and increased healthcare costs.^(4–9)^ Healthcare institutions in recent years have begun to screen for food insecurity and provide resources to address the issue, and food insecurity screening is also often part of mandatory social determinants of health screens in certain institutions and U.S. states.^(10)^ Food insecurity alleviation programmes at healthcare institutions have the potential to provide food and food-related support services directly to their patients. Provision of food not only impacts dietary intake and alleviates food insecurity, but also can free up food dollars to be spent on other necessities, such as housing, transportation, utilities, or medications.^(11)^ Food insecurity programmes implemented at healthcare institutions have previously found positive impacts on food security,^(12)^ diet quality,^(13–17)^ cardiometabolic biomarkers,^(15)^ hospital admission and readmission rates,^(18)^ and healthcare costs.^(19)^ They can also connect patients to resources that can alleviate food insecurity^(20,21)^ and improve management of chronic conditions and health outcomes.^(22)^ Healthcare-based food insecurity programmes are likely to be more successful if they are consistent and sustainable.^(23)^

There is a growing literature base regarding healthcare-based food insecurity programmes.^(24,25)^ Nonprofit and advocacy organisations, such as Feeding America, the Food Research & Action Center, and Children's HealthWatch, have established guidelines on creating and executing these programmes.^(26)^ However, there is a lack of specific, detailed, and actionable implementation guidance for healthcare institutions of differing sizes and types to design, operate, and maintain an array of food insecurity alleviation programmes. Healthcare providers have also expressed that education and assistance with logistics would facilitate implementation of food insecurity alleviation programmes.^(27)^

As a result of United States Department of Agriculture (USDA) funding, there is a small, but growing, evidence base of evaluations of produce prescription programmes,^(28)^ a type of food insecurity intervention located at healthcare institutions in which a provider writes a ‘prescription’ for fruits and vegetables.^(29)^ However, produce prescription programmes may not be the right intervention for every healthcare institution and its patient population.

To support decision-makers and implementers, we aimed to create a typology of intervention components that can be used to create and execute impactful food insecurity programmes at healthcare institutions. We conducted a scoping review of food insecurity interventions based at healthcare institutions and used content analysis^(30)^ to create a typology of programmes. This typology may assist programme implementers consider programmatic intended impact, institutional logistical constraints, and planning for sustainability in an effort to effectively achieve their goals.

## Methods

**Fig. 1.**
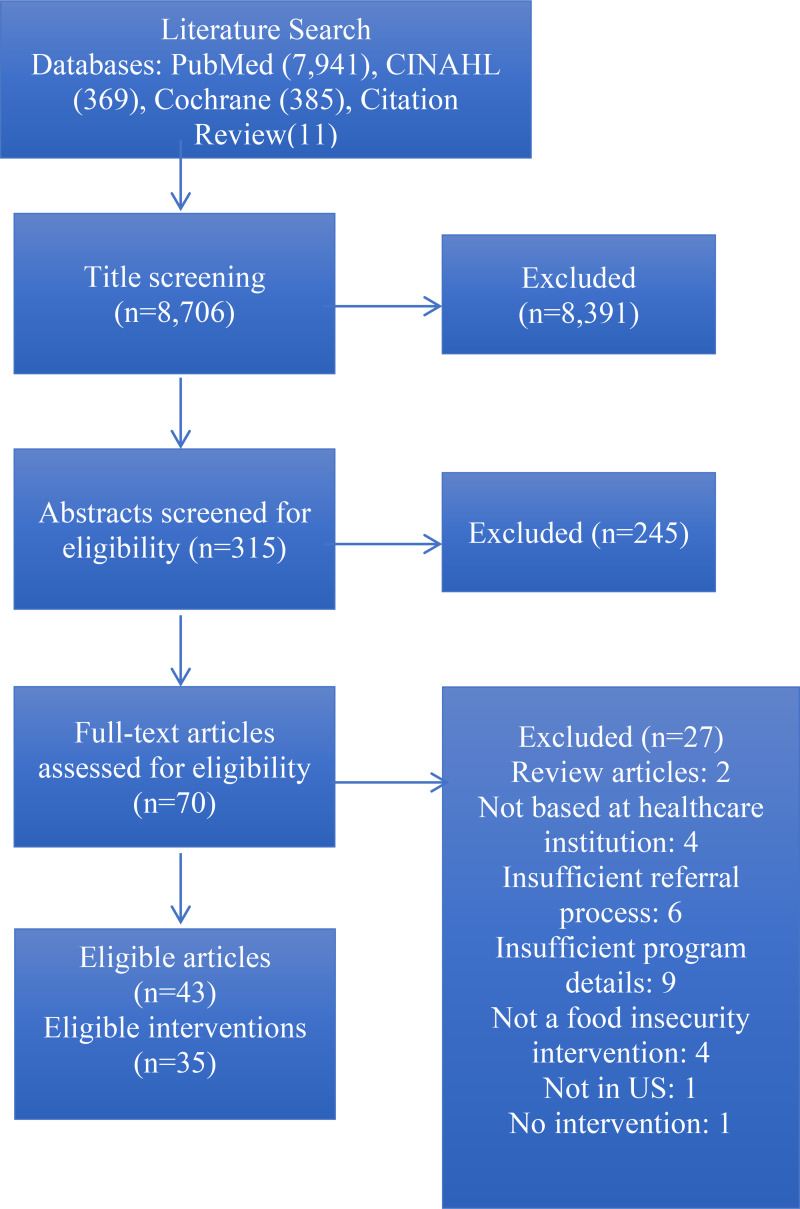
This PRISMA flow diagram depicts our systematic search process that we followed in order to identify articles for inclusion in this work.

We searched PubMed, CINAHL, and Cochrane databases for papers published between 1 January2010 and 31 December2021, with the following terms: food assistance, initiative, program, health, medical, medical center, academic, community health, federally qualified, and food. We also mined reference lists of relevant research articles. We included peer-reviewed papers of interventions based at healthcare institutions in the United States that provided food assistance for their patient population. Papers were eligible for inclusion if they reported implementation and intervention details and patient population descriptions. We included papers that did not evaluate outcomes as long as details regarding programme design and implementation were included. We excluded interventions that screened for food insecurity without providing assistance obtaining food, and those that used a passive referral process, such as handing out a list of available resources, as these interventions have been found to have minimal success connecting patients to resources.^(31)^ Our search strategy is summarised in Fig. 1.

After identifying the manuscripts eligible for inclusion in the review, we created a data charting form with intervention component categories previously identified in the literature. Intervention components included the location of the program, type of healthcare institution in which the programme was located, an overview of the program, duration of patients’ participation in the program, and patient eligibility for the programme. Two reviewers, R.R. and E.M., independently read through each complete manuscript and completed the chart by extracting relevant programmatic information and performing a content analysis.^(30)^ Reviewers met regularly throughout the data extraction process to compare completed charts, discuss discrepancies, and create consensus. Any discrepancies were moderated by J.G.

We then analysed programme components and created the typology. We iteratively compared and contrasted different programme components to identify patterns and groupings that often were often observed together or seemed to influence other programme elements. We also compared any overlapping components or patterns found in multiple categories to further explore these classification definitions, identifying distinguishing characteristics. We continued this process through five iterations until each category had a distinct set of characteristics, resulting in a draft typology. Finally, we validated the draft typology against the research articles to ensure accuracy, with each article fitting distinctly into each category.

## Results

Among 8706 identified articles, forty-three met inclusion criteria. Several articles reported on the same intervention; ultimately, thirty-five distinct interventions were represented in the forty-three articles identified for inclusion. They are summarised in Table 1.

**Table 1.** Description of Healthcare-based food assistance programmes

First author, year	Location in U.S.	Type of healthcare institution	Programme overview	Programme participation duration	Patient eligibility
Adams, 2021^(32)^ & Dunn, 2021^(33)^	Revere, MA	Community Healthcare system	Free produce market	Ongoing	No eligibility requirements
Aiyer, 2019^(34)^	North Pasadena, TX	Federally qualified health center	Fruit and vegetable prescription program	6 months	18+, Food insecure based on two-question clinic screener, residing in one of three target zip codes
Beck, 2014^(35)^	Cincinnati, OH	Urban academic pediatric primary care clinic adjacent to Cincinnati Children's Medical Center	Screening, Provision of Infant Formula, Referral	Ongoing	Families with children <12 months with food insecurity (answered yes to at least one of two screening questions), or failure to thrive. Clinical provider given latitude to deem eligible.
Berkowitz, 2021^(36)^ & Xie, 2021^(37)^	North Carolina	Nine primary care clinics (primarily Federally qualified health centers)	SNAP subsidy for fruits and vegetables	6 months	18+, current SNAP beneficiaries and identified by staff at primary care clinic as ‘likely to benefit from programme b/c of health status’
Blitstein, 2020^(38)^	‘Large Midwest City’	Federally qualified health centers	Screening, referral, nutrition education, enrollment assistance	Ongoing	Diagnosis of diabetes [All participants received intervention regardless of food insecurity status]
Bryce, 2017^(39)^	Detroit, MI	Federally qualified health center	Fruit and vegetable prescription program	13 weeks	FQHC patients with diabetes mellitus, hypertension, obesity, hyperlipidaemia, pregnant or with children, and have limited access to fruits and vegetables
Cavanagh, 2017^(40)^	Upstate NY	Community clinic	Fruit and vegetable prescription program	13 weeks, can receive additional 13 weeks if all coupons used	Low income persons with at risk or with obesity, hypertension, or diabetes
Cohen, 2017^(41)^ & 2019^(42)^	Ypsilanti, MI	Ypsilanti Health Center primary care clinic	Waiting room intervention encouraging Double Up Food Bucks use	One-time	18+, currently SNAP enrolled, self-identified as a primary food shopper for household
Cook, 2021^(43)^ & Newman, 2021^(23)^	Atlanta, Athens, Augusta, GA	Primary care clinics	Produce Prescription programme with nutrition education	6 months	SNAP-eligible and/or screen positive for food insecurity on USDA 2-item screener and diagnosed for one or more condition: overweight/obesity, diabetes mellitus, pre-diabetes, hypertension, or hyperlipidaemia
Esquivel, 2020^(44)^	Rural community near Honolulu, HI	Federally qualified health center pediatric clinic	Fruit and vegetable prescription program, on-site farmer's market (pre-existing)	3 months	Children aged 2–17 with ‘poor nutrition’ based on growth assessment or BMI% < 5 % or >85 %.
Ferrer, 2019^(15)^	San Antonio, TX	Primary care practice	Nutrition education and free box of food	6 months	A1c > 9 %, screen positive for food insecurity on Hunger Vital Sign
Gany, 2020^(12)^	NYC, NY	Outpatient Cancer Clinics	Co-located medically-tailored food pantries at clinics	Throughout treatment/Ongoing	Screen positive for food insecurity on USDA household food insecurity survey
Ghouse, 2020^(45)^	Michigan	Primary care clinic	Nutrition education and free bag of food	6 weeks	Pre-diabetic (A1c 5⋅7–6⋅4 %), screen positive for food insecurity using USDA 5-item, able to converse in English
Greenthal, 2019^(46)^	Northeastern US	Academic urban safety-net hospital	Medically-tailored food pantry	Ongoing	Patients who screen positive for food insecurity in hospital's outpatient clinics
Hager, 2020^(47)^	Minneapolis, MN	Safety-net Medical Center hospital, outpatient clinics, and community primary care clinics	EMR Hunger Vital Sign screening and auto-faxed referral to food bank partner, food bank calls patient to provide assistance with obtaining food assistance in the community	One-time	Screen positive for food insecurity on Hunger Vital Sign
Izumi, 2020^(48)^	Multnomah County, OR	Federally qualified health center safety-net clinic	Subsidised Community-Supported Agriculture	23 weeks	English and Spanish-speaking who receive services at the FQHC.
Jones, 2020^(49)^	Navajo Nation, NM	19 HC facilities in Navajo Nation (range from small clinics to hospitals)	Fruit and vegetable prescription program	6 months	Families with pregnant woman or children </=6 years. Some sites used Indian Health Services Food Insecurity Screening Questionnaire to specifically enroll food insecure families.
Knowles, 2018^(50)^	Philadelphia, PA	Academic medical center pediatric primary care outpatient clinics	Screening, Referral, Benefits eligibility screening and application assistance	One-time	Pediatric patients of center clinics who screen positive for food insecurity
Kulie, 2021^(51)^	Washington, DC	Urban academic Emergency Department	Screening, Referral	One-time	18+, non-life threatening emergency severity index score, insured by DC Medicaid and approved for ED discharge, English-speaking
Milliron, 2017^(52)^	North Carolina	Urban outpatient clinic (free or reduced price services)	Community garden	Ongoing	None
Mirsky, 2021^(53)^	Revere, MA	Community-based academic medical center	On-site plant-based food pantry	12 weeks* original plan that was altered due to COVID-19 in March 2020	Food insecure with diagnosis of obesity, hypertension, or diabetes mellitus. Pilot pts were English-speaking only.
Morales, 2016^(54)^	Chelsea, MA	Community health center	Screening, enrollment assistance and pantry info	One-time	Pregnant women 18+ receiving care at the health center
Paolantonio, 2020^(55)^	NYC, NY	Four cancer clinics	Food voucher program	6 months	18+, able to answer survey questions in English or Spanish, living independently, score low or very low for food insecurity on the USDA Household Food Security Screener, be within 2 weeks of starting radiation or 1 month of starting chemo. Those receiving or applying for SNAP were ineligible.
Sastre, 2021^(18)^	Greenville, NC	Academic medical center inpatient	Hospital-based Medical Food Pantry	One-time	Screen positive for food insecurity on Hunger Vital Sign
Saxe-Custack, 2018^(56)^ & 2019^(57)^	Flint, MI	University-affiliated residency training pediatric clinic	Fruit and vegetable prescription program	Ongoing	All pediatric patients of practice [all receive Rx regardless of need]
Schlosser, 2019a^(58)^; Schlosser, 2019b^(59)^; & Joshi, 2019^(60)^	Cuyahoga County, OH	Three safety-net clinics	Produce Prescription Program	3 months	Patient of clinic positive for food insecurity on 2-item screen and diagnosis of hypertension
Slagel, 2021^(61)^	Athens, GA	‘Clinics’	Produce Prescription programme with nutrition education	6 months	18+, SNAP-eligible or otherwise underserved, with a dx of at least one condition: overweight/obesity, diabetes mellitus, pre-diabetes, hypertension, or hyperlipidaemia
Smith, 2017^(62)^	San Diego, CA	Student-run free clinic associated with medical school	Food Insecurity Screening and Referral with federal programme sign-up assistance, food provided only for patients with DM	One-time; *Patients with diabetes received food bag monthly for unknown amount of time	18+ and patient of student-run free clinics
Stenmark, 2018^(63)^	Colorado	Two pediatric clinics	Screening, Referral, Assistance signing up for fed asst programmes	One-time	Patient of clinic, screen positive for food insecurity on Hunger Vital Sign
Stotz, 2019^(64)^	Georgia	Safety-net clinic	Nutrition education, 12-week receipt of one bag of locally-grown produce	12 weeks	Adults eligible for SNAP-Ed living in households below 185 % federal poverty level (as evidenced by enrollment in safety-net clinic) and diagnosis of diabetes mellitus, hypertension, &/or hyperlipidaemia
Veldheer, 2021^(65)^	Reading, PA	Primary care clinic in community-based hospital	Fruit and vegetable prescription program	7 months	A1c >= 7 %, adults >18 years, BMI >= 25
Walker, 2021^(66)^	‘Large metropolitan region in Ohio’	Two community-based family medicine practices within an academic medical center	Screening, Referral, Vouchers	90 days	18+, patients of primary care clinics, screen positive for food insecurity, and meet clinical criteria, including diagnosis of diabetes mellitus, obesity, hypertension, HgbA1c > 9 %, or pregnant w/ gestational diabetes
Weinstein, 2014^(67)^	Bronx, NY	Primary care or diabetes clinics at urban public hospital	Nutrition education and food voucher	One-time	>18 years and established patient on clinics, with diagnosis of type 2 diabetes mellitus, BMI > 25, A1c > 7 %
Wetherill, 2018^(68)^	Oklahoma	Two academic medical center-affiliated free clinics	Nutrition education and free box of food	6 months	Any patient of affiliated clinics
Wynn, 2021^(63)^	Cook County, IL	Large urban academic medical center – three inpatient units for pilot	Screening for SDOH, paper referral, bag of non-perishable food	One-time	Inpatient on participating floors, screen positive for food insecurity on Hunger Vital Sign

### Results of content analysis

In our content analysis, we identified eight core characteristics of healthcare-based food insecurity programmes: screening for food insecurity, defining eligibility criteria, direct provision of food, provision of vouchers, provision of referrals, offering patient education, healthcare team involved in staffing, and programme length. These characteristics were consistent with the literature, used to assess the forty-three articles in our review, and informed the creation of the typology.

#### Screening for food insecurity

Eighteen^(12,15,18,19,23,34,35,38,43,45,47,50,53,55,58,59,60,62,63,66,69)^ of the thirty-five programmes discussed screening for food insecurity. Of these, six programmes^(23,38,43,45,55,62,70)^ used the USDA Household Food Security Survey, with number of items ranging from two to eighteen. Thirteen programmes^(15,18,34,35–37,47,49,50,53,58–60,63,66,69)^ screened for food insecurity using the Hunger Vital Sign.,^(71–73)^ two programmes^(41,42,51)^ used unique screeners, and one used a ‘standardised assessment form’.^(54)^ programmes that did not screen for food insecurity were either open to anyone regardless of food insecurity status,^(32,33,52)^ open to patients of clinics that serve primarily low-income populations, or used medical records to identify patients with ‘poor nutrition’.^(44)^ One programme used the clinical expertise of an on-site nutritionist to identify eligible patients.^(40)^

#### Defining eligibility criteria

Sixteen^(15,23,36–40,43,44,45,53,55,58,59,60,61,64–67)^ of the thirty-five programmes included a cardiometabolic diagnosis (e.g. overweight, obesity, pre-diabetes, diabetes mellitus, hypertension, or hyperlipidaemia) as part of their eligibility criteria. One programme^(54)^ was created explicitly for pregnant women, four^(35,44,50,57)^ for children, and one^(49)^ for families with either pregnant women or children under age seven. Fourteen^(12,15,18,34,35,45–47,50,53,55,58–60,63,69)^ programmes required patients to screen positive for food insecurity in order to be enrolled. One programme^(23,43)^ required patients to be eligible for the Supplemental Nutrition Assistance Programme (SNAP) or to screen positive for food insecurity.

#### Direct provision of food

Fourteen programmes^(15,18,32,33,35,45,46,48,52,53,55,62–64,68)^ provided food directly to patients. Of these, eight^(12,15,18,45,46,48,53,68)^ provided both produce and non-perishable foods, three^(32,33,52,64)^ provided only produce, one [40] provided only non-perishable foods, and one^(35)^ provided baby formula. Refrigerated storage capacity was noted as a barrier to providing produce directly to patients.^(12,53)^ programmes circumvented this issue by partnering with community-based organisations (CBOs) to provide same-day delivery and distribution of produce,^(12,32,33,48,64)^ while others obtained dedicated refrigeration space.^(46,53)^ One programme^(52)^ utilised an on-site community garden. Two programmes ^(12,46)^ that provided food directly to patients offered a choice of food, while ten programmes^(15,18,32,33,45,48,52,53,63,64,68)^ provided participants with a pre-packed bag. Three programmes ^(12,18,46)^ provided medically-tailored foods, that is, foods that met the nutritional requirements of the patient based on their medical status, as designated by their physician or registered dietitian. These three medically-tailored programmes operated as on-site food pantries.

#### Provision of vouchers

Fourteen programmes ^(23,34,36,37,39,40–44,49,55–62,65–67)^ provided vouchers or other cash incentives that allowed participants to increase their purchasing power for food. By virtue of providing cash assistance to purchase foods, all fourteen programmes provided the participant with some form of choice in the foods they received. None of these programmes provided medically-tailored foods.

The amount of money provided to participants in voucher programmes ranged from a minimum of $6 for the entire programme in 2011 dollars^(67)^ to a maximum of $230 per month for six months in 2021 dollars.^(55)^ Four programmes ^(23,43,49,61)^ provided $1 (between 2015 and 2018) per household member per day.

Ten programmes^(23,39,40–44,56–61,65,67)^ allowed the vouchers to be utilised only for fresh fruits and vegetables, and three programmes^(34,36,37,49)^ allowed voucher use for both fresh and non-perishable healthful (e.g. fruits, vegetables, or whole grains, etc.) foods. One programme allowed participants to use the voucher for any food purchases, though they were reminded to choose healthful foods.^(55)^ Ten programmes^(23,39,41–44,49,56,57–61,65,67)^ partnered with farmer's markets to accept the vouchers, two^(39,44)^ of which were markets located on-site at the healthcare center where the programme was implemented. One programme^(34)^ provided vouchers for a food pantry, and one programme^(40)^ provided a voucher to be used at a mobile produce truck that parked at the health center where the intervention was implemented once weekly. Three programmes^(36,37,49,55)^ allowed participants to utilize the programme at participating partner supermarkets, and one^(49)^ at convenience stores. Three^(39,49,55)^ used debit cards and one programme^(36,37)^ loaded additional funds onto EBT cards. Others used printed vouchers or tokens.

Nine programmes^(23,34,39,43,44,49,56–58,60,61,65)^ were fruit and vegetable prescription (FVRx) programmes, in which clinicians wrote ‘prescriptions’ that patients could take to farmer's markets to purchase a certain number of fruits and vegetables.

#### Provision of referrals

Thirteen programmes^(12,18,35,38,45,50,51,54,62,63,66,69,71)^ provided referrals to local or national food programmes, such as local food pantries, SNAP, or the Special Supplemental Nutrition programme for Women, Infants, and Children, commonly referred to as WIC. Of these, twelve^(18,35,38,45,50,51,54,62,63,66,69,71)^ of these programmes referred to local CBOs, such as food pantries, and eight^(12,35,38,50,54,62,69,71)^ assisted with enrollment in SNAP and WIC. Seven programmes^(12,35,38,50,54,62,69,71)^ provided both. Six programmes^(18,38,51,54,62,66)^ utilised on-site benefits specialists to assist with referrals, while five^(35,50,63,69,71)^ used electronic referrals to alternative CBOs (e.g. food bank,^(66)^ NowPow,^(63)^ Benefits Data Trust,^(50)^ medical–legal partnership,^(35)^ or Hunger Free Colorado^(69)^), who then followed up with the patient.

#### Offering patient education

Nineteen programmes ^(15,18,23,34,35,38,39,43,45,46,48,49,52,53,58–61,64,65,67,68)^ provided education to patients, which included nationally-available courses such as Cooking Matters^(45,74)^ and ‘Eat Right When Money's Tight’,^(38)^ as well as education produced by the intervention healthcare institution. Of the 19, sixteen programmes ^(15,18,23,34,35,38,43,45,49,52,53,58–61,64,65,67,68)^ provided nutrition education, while eight^(23,39,43,45,46,48,49,61,64)^ provided cooking demonstrations or education. Five programmes^(23,43,45,49,61,64)^ provided both. Seven of the nineteen programmes^(15,23,35,38,43,58,59,60,64,68)^ provided education on cooking on a budget or food resource management. Six programmes^(34,35,38,58,59,64,68)^ used passive nutrition education, such as with printed materials or online videos, while nine^(15,23,39,43,45,46,52,53,61,65)^ provided in-person education or cooking demonstrations. Two programmes^(48,49)^ provided both printed materials and in-person cooking demonstrations. Four programmes^(39,48,52,53)^ provided education informally, such as during other healthcare visits,^(52)^ in passing and without billing,^(53)^ or during a farmer's market.^(39,48)^

### Health care team members involved in staffing

Staffing of interventions was varied. Screening and/or referral into interventions was completed by clinic staff in eight programmes,^(12,34,36,37,48,49,65,68,69)^ by a physician in three programmes,^(44,47,67)^ by a programme manager in one program,^(53)^ by a nutritionist in one,^(40)^ and by research study staff in four programmes.^(41,42,50,51,55)^ Seven other programmes listed ‘providers’ as tasked with screening and/or referring patients into the program, but did not specify which type of provider.^(23,38,39,43,46,52,54,58,60)^

Programming was staffed by an array of volunteers and professionals. While some programmes had only one type of staff member running programming, other programmes used a variety of staff to execute different functions of the program, in addition to their primary responsibilities. Community volunteers were utilised by three programmes,^(32,33,34,52)^ while staff volunteers were used by one.^(63)^ CBOs delivered programming in seven interventions.^(15,35,50,51,62,66,69)^ A range of medical staff was used in programme delivery, such as for education: four programmes used providers,^(34,35,49,57,67)^ one used Registered Nurses,^(18)^ one used Nurse Practitioners,^(61)^ five used Registered Dietitian Nutritionists,^(18,40,61,65,68)^ and three used Community Health Workers.^(15,48,65)^ One programme utilised a nutrition educator and licensed chef to deliver education.^(45)^ Research staff delivered programming in one intervention.^(62)^

#### Programme length

Eight programmes^(12,32,33,35,46,52,53,56,57,62)^ were permanent. These programmes included on-site food pantries^(46,53,55)^ and gardens,^(52)^ two monthly on-site food distributions,^(32,33,62)^ on-site distribution of baby formula,^(35)^ and a produce prescription programme.^(56,57)^ Eleven programmes^(18,38,41,42,47,50,51,54,62,63,67,69)^ included programming that each patient received only once, even if they attended the healthcare institution repeatedly. All of these programmes, except one,^(67)^ included food insecurity screening and referral to federal or local food assistance programmes. Two of these programmes also provided participants with one bag of non-perishable foods from a hospital-based food pantry,^(18,63)^ and one included ongoing monthly on-site food distributions to participants with diabetes.^(62)^ Eighteen^(15,23,34–37,39,40,43,44,48,49,55,58–61,64–66,68)^ programmes were time-limited, ranging from three to nine months. Fourteen^(23,34–37,39,40,43,44,49,55,58–61,65,66)^ of these programmes utilised cash assistance to increase participants’ purchasing power of food, while four^(15,48,64,68)^ provided food directly to patients.

### Results of typology development

We identified three types of food assistance programmes located at healthcare institutions: those that provide food directly to patients, those that refer patients to resources that provide food, and those that provide vouchers or cash assistance in order to purchase food. These are summarised in Table 2.

**Table 2.** Typology of healthcare-based food assistance programmes

	Provides food directly	Refers to resources that provide food assistance	Provides vouchers to purchase food
Recipients	Patients who are both food insecure and have a diagnosis of chronic disease (e.g. diabetes mellitus, cardiovascular disease, or cancer, etc.). Sometimes participants with food insecurity and no additional eligibility requirements.	All patients who screen positive for food insecurity	Patients who are both food insecure and have a diagnosis of cardiometabolic disease (e.g. diabetes mellitus or cardiovascular disease, etc.) or health status associated with nutritional risk (e.g. pregnancy, obesity, or underweight, etc.)
Length of Intervention	Ongoing, allows for repeat use by participants.	One-time intervention to connect people to resources	Time-bound, providing resources for an average of 3–6 months
Type of Food	Both produce and non-perishable foods	No type of food prioritised (referral to programming, regardless of food provided)	Priority to provide produce (fruits and vegetables)
Choice of Food	Limited choice, if any. Typically offer standard bags of food that do not provide patient with choices	No tangible food provided	Moderate choices offered. Allow participants to choose foods within certain parameters (e.g. fruits and vegetables)
Education	Typically provides nutrition education, sometimes with cooking demonstrations included	No education provided	Often provides nutrition education, sometimes includes cooking demonstrations
Partnerships	Partnerships with CBOs to obtain food to be distributed.	No formal partnerships required. Some programmes set up direct lines to local CBOs (e.g. food pantries, food banks, Feeding America franchise).	Partnerships with food purveyors (e.g. farmers, grocery stores) to accept vouchers.
Reach	Varied. Can be limited in size or available to a large proportion of patients (in the case of on-site gardens, pantries)	Significant. Available to a large proportion of patients. Successful reach may be dependent on capacity of referred CBOs.	Limited in size
Staffing	Referral and enrollment process facilitated by varying clinic staff members. Two programmes were entirely open access.	No permanent staff focused on this programming. Often filled though research staff roles	Often require a physician to refer patient into programme

#### Programmes that provide food directly

programmes that provide food directly to patients provide both produce and non-perishable foods and tend not to offer patients a choice of food, instead, providing a standardised pre-packed bag or box. These programmes frequently include nutrition education, and sometimes cooking demonstrations, and tend to be permanent and on-site. Examples include food pantries, community gardens, and on-site food delivery and distribution in partnership with a local food bank. Often, institutions and programmes partner with CBOs for obtaining the food to be provided.

Patients can be referred to these programmes by their healthcare providers (e.g. physicians or dietitians) and utilize the programme at any time at any time in their care, as they are static programmes. These programmes vary in size: some provide assistance to a limited number of patients, while others provide food to any patient who would benefit. Often these programmes are only developed for and provided to patients with certain diagnoses, most often nutrition-related cardiometabolic diagnoses, such as diabetes, obesity, and cardiovascular disease. However, it is not uncommon that these programmes also provide foods for all patients with food insecurity, regardless of cardiometabolic diagnosis.

#### Programmes that refer patients to resources that provide food assistance

Programmes that refer to resources that provide food assistance are typically available for all patients that screen positive for food insecurity, either on the Hunger Vital Sign^(71,72,73)^ or another screening tool.^(75)^ These programmes can be time-limited or permanent at the healthcare institution, but each participant's interaction with the programme happens only once, when they are referred to resources.

Referrals can be to local CBOs such as food pantries, regional resources such as a Feeding America site, or enrollment assistance with federal food assistance programmes such as SNAP and WIC. Enrollment assistance happens both on-site or via referral to a CBO to assist.

Referral programmes are more limited in scope than other food assistance programmes. Because the programme consists of a referral to an outside entity, the healthcare institution has no control over the type of food provided; patients instead receive food assistance from the CBO or federal programme. Nutrition education is rarely provided by the healthcare institution in tandem with the referral programme. Reach for these programmes is significant; many patients are able to be referred. The referral programmes we identified used research staff to complete the patient identification and referral process. Use of temporary research staff, rather than permanent healthcare staff, highlights the uncertainty of programme funding and sustainability.

#### Programmes that provide vouchers to purchase food

Programmes that provide vouchers to purchase food are time-bound, providing patients with vouchers for an average of three to six months. They are typically available for patients with both food insecurity and a health status associated with nutritional risk (e.g. pregnancy obesity, underweight) or cardiometabolic disease. Produce prescription programmes, which are a type of voucher program, are frequently funded by the USDA National Institute of Food and Agriculture Gus Schumacher Nutrition Incentive programme (GusNIP), which requires that they reach low-income populations with diet-related health conditions.^(29)^

Voucher programmes prioritize the procurement of fruits and vegetables over non-perishable foods and provide a choice of foods within this limitation (i.e. patients can choose which fruits or vegetables they would like to purchase with their voucher). Nutrition education and cooking demonstrations are frequently paired with these programmes. Partnerships with food purveyors, such as farmers, farmer's markets, supermarkets, and convenience stores, are necessary for providing a venue in which the vouchers are accepted.^(29,76)^ These programmes are often limited in reach, helping fewer patients than other types of programmes, likely due to the cost of providing financial assistance. These programmes often require a physician to refer the patient to the program; they are rarely open to all patients; only those with a prescription can participate.

## Discussion

We used results of our scoping review to create a typology that identified three distinct types of healthcare-based food insecurity interventions: those that provide food directly to patients, those that refer patients to resources that provide food assistance, and those that provide vouchers to purchase food. Our findings from the typology indicate that logistical considerations and constraints impact feasibility of healthcare-based food insecurity interventions. Important logistics to consider include staffing, refrigeration and storage space, existence of willing CBOs and partners, and programme goals.

Staffing of food insecurity alleviation programmes varied by the type of programme: programmes that provide vouchers or financial assistance to patients often require a physician to refer qualifying patients to the programme, while programmes that provide food directly to patients utilize a variety of clinic staff members, such as registered dietitians and community health workers. Referral programmes rarely used permanent staff, and often filled these positions with members of the research team.

Implementation science literature indicates that staffing of healthcare-based programming heavily influences implementation and sustainability.^(77)^ Analysing staff capacity prior to programme development and implementation and aligning programme choice with existing capacity should help to ensure that adequate human resources are in place. Physicians have previously reported that limited training and time during patient visits are barriers to implementing voucher programmes.^(28)^ If adequate training and time is unavailable, implementers may wish to consider a programme that does not require physician involvement, such as a referral programme. Importantly, having a dedicated, paid, staff member, rather than a volunteer programme champion, to run any type of food insecurity alleviation programme improves provider experiences and overall programming.^(28)^

Refrigeration and storage space for food provision also heavily impacts the type of programming a healthcare institution can implement. For example, many of the programmes we included in the review lacked refrigeration space for storing fresh produce; some programmes chose to ameliorate this issue by providing only non-perishable foods, while others partnered with CBOs to deliver fresh produce to be distributed to patients on the same day. Securing space agreements and identifying community partners early in programme development not only help to increase likelihood of programme success but also dictate what type of programme may best fit the needs of the healthcare institution and patient population.^(53)^

Community partnerships were influential in almost all the programmes we identified. Programmes that provided food directly to patients often partnered with local food banks to source the food; referral programmes partnered with CBOs to assist with registering patients for SNAP and WIC, or to local food pantries for additional food resources; and programmes that provided vouchers to patients often partnered with food purveyors such as farmers markets or supermarkets. Identifying and working with community partners early in development and implementation of food assistance programmes will dictate what programming can be provided effectively.^(29,53,69)^.

In addition to logistical constraints, healthcare institutions should consider their goals for the program, including how many and what type of patients they aim to reach and the length of time of the intervention they foresee. As evidenced in the typology, programmes that wish to serve a greater number of food insecure patients may wish to implement a referral program, which allows for higher throughput than a voucher or food provision programme.^(69,71)^ Alternatively, institutions that wish to provide programming for patients with the dual burden of food insecurity and cardiometabolic disease may choose to implement a more rigorous, but less wide-reaching initiative, such as a fruit and vegetable prescription programme.^(29)^ It is, however, important to consider the length of the intervention; provision of food for a months-long period is a worthy goal but may lack durability of any health or food security outcomes observed.^(23)^

A limitation of this typology is that we do not include information on programme sustainability. Healthcare institutions that aim to create food insecurity alleviation programmes should consider not only their implementation, but their plan for sustainability. Research indicates that sustainability of healthcare-based programmes is heavily influenced by organisational support, staff turnover, and funding.^(23,77)^ Being explicit about programme components and clearly defining scope of work and processes can increase sustainability and achievement of strategic outcomes.^(78)^ Referral programmes may be best able to reach the largest number of patients, but follow-through to ensure that patients are connected to and receive resources is a challenge.^(24)^

A second limitation to this typology is that we did not assess programme outcomes, but rather the implementation and programming. Importantly, a 2019 review of healthcare-based food insecurity interventions found that the majority of studies were low quality and most analysed only process outcomes.^(24)^ Evaluation of programme impacts and outcomes, such as improvements in health or decreased healthcare utilisation, can lead to increased funding, and thus sustainability, of these programmes.^(28)^ Formal evaluations are often not done, however, because many of these programmes are small in scope and created by clinical staff members to help patients, rather than researchers aiming to evaluate a programme.^(79)^ Additionally, it is difficult to assess the body of literature as a whole because of the heterogeneity of programme components^(24,79^^)^ and goals: some to improve food security and some to improve overall health and healthcare spending. Future research should aim to identify which programme components and outcomes are most important for improving food insecurity among patient populations.

Lastly, this scoping review and typology included only interventions from the peer-reviewed literature; there are certainly other healthcare-based food insecurity programmes in the United States that have not published peer-reviewed literature of their findings.^(22,25,80)^ There is likely significant institutional knowledge at other sites, and the field would benefit from tapping into both the positive and negative experiences of existing programmes. Communities of practice, focused forums, and other forms of information sharing may be the best way to identify learnings and innovations, and ultimately share effective practices.

## Supporting information

Rudel et al. supplementary material Rudel et al. supplementary material

## Supplementary material

The supplementary material for this article can be found at https://doi.org/10.1017/jns.2023.111

## References

[1] Feeding America (2021) The Impact of the Coronavirus on Food Insecurity in 2020 & 2021. Accessed November 2021. https://www.feedingamerica.org/sites/default/files/2021-03/National%20Projections%20Brief_3.9.2021_0.pdf.

[2] United States Department of Agriculture (2021) Definitions of Food Security. Accessed March 2022. https://www.ers.usda.gov/topics/food-nutrition-assistance/food-security-in-the-u-s/definitions-of-food-security/.

[3] Coleman-Jensen A, Rabbitt MP, Gregory CA & Singh A. Household Food Security in the United States in 2021, ERR-309. U.S. Department of Agriculture, Economic Research Service; 2022.

[4] Seligman HK, Davis TC, Schillinger D & Wolf MS. Food insecurity is associated with hypoglycemia and poor diabetes self-management in a low-income sample with diabetes. J Healthcare Poor Underserved. 2010;21(4):1227–1233, 10.1353/hpu.2010.0921. PMC3004431 21099074

[5] Allen NL, Becerra BJ & Becerra MB. Associations between food insecurity and the severity of psychological distress among African-Americans. Ethnic Health. 2018;23(5):511–520, 10.1080/13557858.2017.1280139. 28140616

[6] Becerra MB, Allen NL & Becerra BJ. Food insecurity and low self-efficacy are associated with increased healthcare utilization among adults with type II diabetes mellitus. J Diabetes Complicat. 2016;30(8):1488–1493, 10.1016/j.jdiacomp.2016.07.009. 27474705

[7] Garcia SP. Incremental health care costs associated with food insecurity and chronic conditions among older adults. Prev Chronic Dis. 2018;15:180058, 10.5888/pcd15.180058. PMC6130288 30171678

[8] Gundersen C & Ziliak JP. Food insecurity and health outcomes. Health Affairs. 2015;34(11):1830–1839, 10.1377/hlthaff.2015.0645. 26526240

[9] Palakshappa D, Garg A, Peltz A, Wong CA, Cholera R & Berkowitz SA. Food insecurity was associated with greater family health care expenditures in the US, 2016–17. Health Affairs. 2023;42(1):44–52, 10.1377/hlthaff.2022.00414. 36623217 PMC10926282

[10] Sandhu S, Sharma A, Cholera R & Bettger JP. Integrated health and social care in the United States: a decade of policy progress. Int J Integr Care. 2021;21(4):9, 10.5334/ijic.5687. PMC8570194 34785994

[11] Berkowitz SA, Shahid NN, Terranova J, et al. ‘I was able to eat what I am supposed to eat’– patient reflections on a medically-tailored meal intervention: a qualitative analysis. BMC Endocr Disord. 2020;20(1):10, 10.1186/s12902-020-0491-z. 31959176 PMC6971854

[12] Gany FM, Pan S, Ramirez J & Paolantonio L. Development of a medically tailored hospital-based food pantry system. J Healthcare Poor Underserved. 2020;31(2):595–602, 10.1353/hpu.2020.0047. PMC8073793 33410795

[13] Liu FX, Alexander GC, Crawford SY, Pickard AS, Hedeker D & Walton SM. The impact of medicare part D on out-of-pocket costs for prescription drugs, medication utilization, health resource utilization, and preference-based health utility. Health Serv Res. 2011;46(4):1104–1123, 10.1111/j.1475-6773.2011.01273.x. 21609328 PMC3165180

[14] Berkowitz SA, O'Neill J, Sayer E, et al. Health center–based community-supported agriculture: an RCT. Am J Prev Med. 2019;57(6, Supplement 1):S55–S64, 10.1016/j.amepre.2019.07.015. 31522922 PMC6874748

[15] Ferrer RL, Neira LM, De Leon Garcia GL, Cuellar K & Rodriguez J. Primary care and food bank collaboration to address food insecurity: a pilot randomized trial. Nutr Metab Insights. 2019;12:1178638819866434, 10.1177/1178638819866434. 31384130 PMC6664622

[16] Freedman DA, Choi SK, Hurley T, Anadu E & Hébert JR. A farmers’ market at a federally qualified health center improves fruit and vegetable intake among low-income diabetics. Prev Med. 2013;56(5):288–292, 10.1016/j.ypmed.2013.01.018. 23384473 PMC3633661

[17] Leone LA, Tripicchio GL, Haynes-Maslow L, et al. Cluster randomized controlled trial of a mobile market intervention to increase fruit and vegetable intake among adults in lower-income communities in North Carolina. Int J Behav Nutr Phys Activ. 2018;15(1):2, 10.1186/s12966-017-0637-1. PMC5756418 29304862

[18] Sastre L, Wynn D, Roupe M & Jacobs M. Link between redemption of a medical food pantry voucher and reduced hospital readmissions. Prev Med Rep. 2021;23:101400, 10.1016/j.pmedr.2021.101400. 34136336 PMC8178117

[19] Berkowitz SA, Terranova J, Randall L, Cranston K, Waters DB & Hsu J. Association between receipt of a medically tailored meal program and health care use. JAMA Intern Med. 2019;179(6):786–793, 10.1001/jamainternmed.2019.0198. 31009050 PMC6547148

[20] Ratcliffe C, McKernan SM & Zhang S. How much does the supplemental nutrition assistance program reduce food insecurity? Am J Agric Econ. 2011;93(4):1082–1098, 10.1093/ajae/aar026. 25197100 PMC4154696

[21] Carlson S & Keith-Jennings B. SNAP Is Linked with Improved Nutritional Outcomes and Lower Healthcare Costs. Washington, DC: Center on Budget and Policy Priorities; 2018. Published January 17, 2018. Accessed January 21, 2022. https://www.cbpp.org/research/food-assistance/snap-is-linked-with-improved-nutritional-outcomes-and-lower-health-care

[22] Feinberg AT, Hess A, Passaretti M, Coolbaugh S & Lee TH. Prescribing Food as A Specialty Drug. NEJM Catalyst; 2018.

[23] Newman T & Lee JS. Strategies and challenges: qualitative lessons learned from Georgia produce prescription programs. Health Promotion Practice. 2021;23(4), 10.1177/15248399211028558. 34416837

[24] De Marchis EH, Torres JM, Benesch T, et al. Interventions addressing food insecurity in health care settings: a systematic review. Ann Fam Med. 2019;17(5):436–447, 10.1370/afm.2412. 31501207 PMC7032918

[25] DAISA Enterprises WW (2021) Produce Prescription Programs US Field Scan Report: 2010–2020. Accessed December 2022. https://www.daisaenterprises.com/uploads/4/4/0/5/44054359/produce_prescription_programs_us_field_scan_report__june_2021_final.pdf.

[26] Children's HealthWatch, Feeding America, and the Food Research & Action Center (2018) Addressing Food Insecurity in Healthcare Settings: Key Actions & Tools for Success. Accessed March 2022. https://frac.org/wp-content/uploads/addressing-food-insecurity-in-health-care-settings-key-actions-and-tools.pdf.

[27] Coward KB, Cafer A, Rosenthal M, Allen D & Paltanwale Q. An exploration of key barriers to healthcare providers’ use of food prescription (FRx) interventions in the rural South. Public Health Nutr. 2021;24(5):1095–1103, 10.1017/S1368980020005376. 33423706 PMC10195487

[28] Stotz SA, Budd Nugent N, Ridberg R, et al. Produce prescription projects: Challenges, solutions, and emerging best practices – perspectives from Healthcare providers. Prev Med Rep. 2022;29:101951, 10.1016/j.pmedr.2022.101951. 36161127 PMC9502043

[29] United States Department of Agriculture (2020). Gus Schumacher Nutrition Incentive Program. Washington, DC: National Institute of Food and Agriculture; 2020. Accessed December 2022. http://www.nifa.usda.gov/grants/programs/hunger-food-security-programs/gus-schumacher-nutrition-incentive-program.

[30] Hsieh HF & Shannon SE. Three approaches to qualitative content analysis. Qual Health Res. 2005;15(9):1277–1288, 10.1177/1049732305276687. 16204405

[31] Marpadga S, Fernandez A, Leung J, Tang A, Seligman H & Murphy EJ. Challenges and successes with food resource referrals for food-insecure patients with diabetes. Perm J. 2019;23:18–097, 10.7812/TPP/18-097. PMC6380483 30939269

[32] Adams E. Providing food assistance during the COVID-19 pandemic: a case study of a free produce market at a health care center. J Healthcare Poor Underserv. 2021;32(4):1–10, 10.1353/hpu.2021.0198. 34803075

[33] Dunn CG, Vercammen KA, Bleich SN, et al. Participant perceptions of a free fresh produce market at a health center. J Nutr Educ Behav. 2021;53(7):573–582, 10.1016/j.jneb.2021.03.012. 34246412

[34] Aiyer JN, Raber M, Bello RS, et al. A pilot food prescription program promotes produce intake and decreases food insecurity. Transl Behav Med. 2019;9(5):922–930, 10.1093/tbm/ibz112. 31570927 PMC6768858

[35] Beck AF, Henize AW, Kahn RS, Reiber KL, Young JJ & Klein MD. Forging a pediatric primary care–community partnership to support food-insecure families. Pediatrics. 2014;134(2):e564–e571, 10.1542/peds.2013-3845. 25049345

[36] Berkowitz SA, Curran N, Hoeffler S, Henderson R, Price A & Ng SW. Association of a fruit and vegetable subsidy program with food purchases by individuals with low income in the US. JAMA Netw Open. 2021;4(8):e2120377, 10.1001/jamanetworkopen.2021.20377. 34379125 PMC8358732

[37] Xie J, Price A, Curran N & Østbye T. The impact of a produce prescription programme on healthy food purchasing and diabetes-related health outcomes. Public Health Nutr. 2021;24(12):3945–3955, 10.1017/S1368980021001828. 33902771 PMC8369461

[38] Blitstein JL, Lazar D, Gregory K, et al. Foods for health: an integrated social medical approach to food insecurity among patients with diabetes. Am J Health Promot. 2021;35(3):369–376, 10.1177/0890117120964144. 33043687 PMC8627420

[39] Bryce R, Guajardo C, Ilarraza D, et al. Participation in a farmers’ market fruit and vegetable prescription program at a federally qualified health center improves hemoglobin A1C in low income uncontrolled diabetics. Prev Med Rep. 2017;7:176–179, 10.1016/j.pmedr.2017.06.006. 28702315 PMC5496208

[40] Cavanagh M, Jurkowski J, Bozlak C, Hastings J & Klein A. Veggie Rx: an outcome evaluation of a healthy food incentive programme. Public Health Nutr. 2017;20(14):2636–2641, 10.1017/S1368980016002081. 27539192 PMC5743436

[41] Cohen AJ, Richardson CR, Heisler M, et al. Increasing Use of a healthy food incentive: a waiting room intervention among low-income patients. Am J Prev Med. 2017;52(2):154–162, 10.1016/j.amepre.2016.11.008. 28109458 PMC5444808

[42] Cohen AJ, Oatmen KE, Heisler M, et al. Facilitators and barriers to supplemental nutrition assistance program incentive use: findings from a clinic intervention for low-income patients. Am J Prev Med. 2019;56(4):571–579, 10.1016/j.amepre.2018.11.010. 30799161 PMC6757336

[43] Cook M, Ward R, Newman T, et al. Food security and clinical outcomes of the 2017 Georgia fruit and vegetable prescription program. J Nutr Educ Behav. 2021;53(9):770–778, 10.1016/j.jneb.2021.06.010. 34509277

[44] Esquivel MK, Higa A, Hitchens M, Shelton C & Okihiro M. Keiki produce prescription (KPRx) program feasibility study to reduce food insecurity and obesity risk. Hawaii J Health Soc Welf. 2020;79(5 Suppl 1):44–49. 32490385 PMC7260871

[45] Ghouse A, Gunther W & Sebastian M. Evaluation of a COVID-influenced curriculum to address food insecurity in a detroit family medicine residency clinic. Spartan Med Res J. 2020;5(2):17649, 10.51894/001c.17649. 33655191 PMC7746036

[46] Greenthal E, Jia J, Poblacion A & James T. Patient experiences and provider perspectives on a hospital-based food pantry: a mixed methods evaluation study. Public Health Nutr. 2019;22(17):3261–3269, 10.1017/S1368980019002040. 31486351 PMC10260686

[47] Hager K, De Kesel Lofthus A, Balan B & Cutts D. Electronic medical record-based referrals to community nutritional assistance for food-insecure patients. Annals of Family Medicine. 2020;18(3):278–278, doi:10.1370/afm.2530. 32393567 PMC7213993

[48] Izumi BT, Martin A, Garvin T, et al. CSA partnerships for health: outcome evaluation results from a subsidized community-supported agriculture program to connect safety-net clinic patients with farms to improve dietary behaviors, food security, and overall health. Trans Behav Med. 2020;10(6):1277–1286, 10.1093/tbm/ibaa041. 33421087

[49] Jones LJ, VanWassenhove-Paetzold J, Thomas K, et al. Impact of a fruit and vegetable prescription program on health outcomes and behaviors in young navajo children. Curr Dev Nutr. 2020;4(8):nzaa109, 10.1093/cdn/nzaa109. 32734135 PMC7377262

[50] Knowles M, Khan S, Palakshappa D, et al. Successes, challenges, and considerations for integrating referral into food insecurity screening in pediatric settings. J Healthcare Poor Underserv. 2018;29(1):181–191, 10.1353/hpu.2018.0012. 29503293

[51] Kulie P, Steinmetz E, Johnson S & McCarthy ML. A health-related social needs referral program for medicaid beneficiaries treated in an emergency department. Am J Emerg Med. 2021;47:119–124, 10.1016/j.ajem.2021.03.069. 33799141

[52] Milliron BJ, Vitolins MZ, Gamble E, Jones R, Chenault MC & Tooze JA. Process evaluation of a community garden at an urban outpatient clinic. J Community Health. 2017;42(4):639–648, 10.1007/s10900-016-0299-y. 27900514 PMC5447497

[53] Mirsky JB, Zack RM, Berkowitz SA & Fiechtner L. Massachusetts general hospital revere food pantry: addressing hunger and health at an academic medical center community clinic. Healthcare. 2021;9(4):100589, 10.1016/j.hjdsi.2021.100589. 34628211 PMC8915928

[54] Morales ME, Epstein MH, Marable DE, Oo SA & Berkowitz SA. Food insecurity and cardiovascular health in pregnant women: results from the food for families program, chelsea, Massachusetts, 2013–2015. Prev Chronic Dis. 2016;13:E152, 10.5888/pcd13.160212. 27809418 PMC5094858

[55] Paolantonio L, Kim SY, Ramirez J, et al. Food purchasing behavior of food insecure cancer patients receiving supplemental food vouchers. Support Care Cancer. 2020;28(8):3739–3746, 10.1007/s00520-019-05183-4. 31828492 PMC8054702

[56] Saxe-Custack A, Lofton HC, Hanna-Attisha M, et al. Caregiver perceptions of a fruit and vegetable prescription programme for low-income paediatric patients. Public Health Nutr. 2018;21(13):2497–2506, 10.1017/S1368980018000964. 29667562 PMC10260820

[57] Saxe-Custack A, LaChance J, Hanna-Attisha M & Ceja T. Fruit and vegetable prescriptions for pediatric patients living in flint, Michigan: a cross-sectional study of food security and dietary patterns at baseline. Nutrients. 2019;11(6):1423, 10.3390/nu11061423. 31242555 PMC6627167

[58] Schlosser AV, Joshi K, Smith S, Thornton A, Bolen SD & Trapl ES. “The coupons and stuff just made it possible”: economic constraints and patient experiences of a produce prescription program. Transl Behav Med. 2019;9(5):875–883, 10.1093/tbm/ibz086. 31570919 PMC6937548

[59] Schlosser AV, Smith S, Joshi K, Thornton A, Trapl ES & Bolen S. “You guys really care about me…”: a qualitative exploration of a produce prescription program in safety Net clinics. J Gen Intern Med. 2019;34(11):2567–2574, 10.1007/s11606-019-05326-7. 31512182 PMC6848686

[60] Joshi K, Smith S, Bolen SD, Osborne A, Benko M & Trapl ES. Implementing a produce prescription program for hypertensive patients in safety net clinics. Health Prom Prac. 2019;20(1):94–104, 10.1177/1524839917754090. 29380633

[61] Slagel N, Newman T, Sanville L, et al. A pilot fruit and vegetable prescription (FVRx) program improves local fruit and vegetable consumption, nutrition knowledge, and food purchasing practices. Health Prom Pract. 2021;24(1), 10.1177/15248399211018169. 34078142

[62] Smith S, Malinak D, Chang J, et al. Implementation of a food insecurity screening and referral program in student-run free clinics in San diego, California. Prev Med Rep. 2017;5:134–139, 10.1016/j.pmedr.2016.12.007. 27990340 PMC5157787

[63] Wynn N, Staffileno BA, Grenier JM & Phillips J. Implementing a food is medicine program to address food insecurity in an academic medical center. J Nurs Care Qual. 2021;36(3):262–268, 10.1097/NCQ.0000000000000496. 32568962

[64] Stotz SA, Thompson JJ, Bhargava V, Scarrow A, Capitano K & Lee JS. A supplemental produce and eLearning nutrition education program for Georgians who use safety-net clinics for their health care. J Nutr Educ Behav. 2019;51(9):1099–1106, 10.1016/j.jneb.2019.06.018. 31345674

[65] Veldheer S, Scartozzi C, Bordner CR, et al. Impact of a prescription produce program on diabetes and cardiovascular risk outcomes. J Nutr Educ Behav. 2021;53(12):1008–1017, 10.1016/j.jneb.2021.07.005. 34426064

[66] Walker D, DePuccio M, Hefner J, et al. Utilization patterns of a clinic-based food referral program: findings from the Mid-Ohio farmacy. Health Serv Res. 2021;56:81–81, 10.1111/1475-6773.13836. 34772772

[67] Weinstein E, Galindo RJ, Fried M, Rucker L & Davis NJ. Impact of a focused nutrition educational intervention coupled with improved access to fresh produce on purchasing behavior and consumption of fruits and vegetables in overweight patients with diabetes mellitus. Diabetes Educ. 2014;40(1):100–106, 10.1177/0145721713508823. 24159007

[68] Wetherill MS, Chancellor McIntosh H, Beachy C & Shadid O. Design and implementation of a clinic-based food pharmacy for food insecure, uninsured patients to support chronic disease self-management. J Nutr Educ Behav. 2018;50(9):947–949, 10.1016/j.jneb.2018.05.014. 30064811

[69] Stenmark SH, Steiner JF, Marpadga S, DeBor M, Underhill K & Seligman H. Lessons learned from implementation of the food insecurity screening and referral program at Kaiser Permanente Colorado. Perm J. 2018;22:18–093, 10.7812/TPP/18-093. PMC6175601 30296400

[70] Gany F. Development of a Medically Tailored Hospital-based Food Pantry System. Published 2020. Accessed October 14, 2021. https://www.ncbi.nlm.nih.gov/pmc/articles/PMC8073793/10.1353/hpu.2020.0047 PMC8073793 33410795

[71] Hager ER, Quigg AM, Black MM, et al. Development and validity of a 2-item screen to identify families at risk for food insecurity. Pediatrics. 2010;126(1):e26–e32, 10.1542/peds.2009-3146. 20595453

[72] Gundersen C, Engelhard EE, Crumbaugh AS & Seligman HK. Brief assessment of food insecurity accurately identifies high-risk US adults. Public Health Nutr. 2017;20(8):1367–1371, 10.1017/s1368980017000180. 28215190 PMC10261547

[73] Cutts D & Cook J. Screening for food insecurity: short-term alleviation and long-term prevention. Am J Public Health. 2017;107(11):1699–1700, 10.2105/AJPH.2017.304082. 29019766 PMC5637690

[74] Cooking Matters. Accessed January 17, 2023. https://cookingmatters.org/

[75] USDA ERS - Survey Tools. Accessed January 31, 2022. https://www.ers.usda.gov/topics/food-nutrition-assistance/food-security-in-the-u-s/survey-tools/#household

[76] Caceres G. Farmers Markets: Local Partners in the Nutrition Incentive Field. Washington, DC: National Institute of Food and Agriculture; 2021. Accessed January 13, 2023. http://www.nifa.usda.gov/about-nifa/blogs/farmers-markets-local-partners-nutrition-incentive-field

[77] Shelton RC, Cooper BR & Stirman SW. The sustainability of evidence-based interventions and practices in public health and health care. Ann Rev Public Health. 2018;39(1):55–76, 10.1146/annurev-publhealth-040617-014731. 29328872

[78] Fiori K, Patel M, Sanderson D, et al. From policy statement to practice: Integrating social needs screening and referral assistance with community health workers in an urban academic health center. J Prim Care Community Health. 2019;10:2150132719899207, 10.1177/2150132719899207. 31894711 PMC6940600

[79] Veldheer S, Scartozzi C, Knehans A, et al. A systematic scoping review of How healthcare organizations are facilitating access to fruits and vegetables in their patient populations. J Nutr. 2020;150(11):2859–2873, 10.1093/jn/nxaa209. 32856074

[80] Feinberg AT, Slotkin JR, Hess A & Erskine AR. How Geisinger Treats Diabetes by Giving Away Free, Healthy Food. Harvard Business Review; 2017. Brighton, Massachusetts. Published online October 25, 2017. Accessed February 24, 2020. https://hbr.org/2017/10/how-geisinger-treats-diabetes-by-giving-away-free-healthy-food

